# Noncovalent Dimerization of Ubiquitin**

**DOI:** 10.1002/anie.201106190

**Published:** 2011-11-23

**Authors:** Zhu Liu, Wei-Ping Zhang, Qiong Xing, Xuefeng Ren, Maili Liu, Chun Tang

**Affiliations:** State Key Laboratory of Magnetic Resonance and Atomic and Molecular Physics, Wuhan Institute of Physics and Mathematics, Chinese Academy of SciencesWuhan, Hubei 430071 (China); Department of Pharmacology, School of Medicine, Zhejiang UniversityHangzhou, Zhejiang (China); National Institute of Child Health and Human Development, National Institutes of HealthBethesda (USA)

**Keywords:** dimerization, NMR spectroscopy, paramagnetic relaxation enhancement, protein–protein interactions, ubiquitin

Ubiquitin is a small signaling protein in cells and is highly conserved throughout the eukaryotes. Ubiquitin interacts with myriad partner proteins that contain one or more ubiquitin-binding domains (UBDs). To achieve multivalent binding with several UBDs, ubiquitins are often covalently linked by an isopeptide bond between the C-terminal carboxyl group of one ubiquitin and a primary amine in another;1a,b the two subunits in a di-ubiquitin are referred to as the proximal unit and the distal unit, respectively. All seven lysines and the N-terminus of ubiquitin can participate in the isopeptide bond.^2^ In tandem, multiple ubiquitins can be linked up to form a poly(ubiquitin).

Depending on the site of the linkage, di- and poly(ubiquitin)s can display distinct quaternary structures, which may account for their linkage-specific functions.1a,b Among the linkages, Lys11, Lys48, and Lys63-linked poly(ubiquitin)s are best characterized: Lys63-linked poly(ubiquitin) is involved in cellular events such as endocytosis and DNA repair, while both Lys11- and Lys48-linked poly(ubiquitin)s can signal for proteosomal degradation.1a,b In crystal, Lys48-linked di-ubiquitin mostly adopts a closed conformation, burying hydrophobic residues around Ile44 in both subunits;3a,b, 4 Lys63-linked di-ubiquitin adopts an open extended structure;5a,b, 6 while Lys11-linked di-ubiquitin displays intermediate subunit separation.^7^, ^8^

Structural heterogeneity has also been observed for di- and poly(ubiquitin)s with the same linkage. Lys48-linked di-ubiquitin has been crystallized in multiple forms, one of which actually adopts an open conformation (Figure S1a),^4^ while in solution at a neutral pH value, approxaimely15 % adopts the open conformation.^9^ Although adopting an extended conformation in crystals,5a,b a highly compact structure was deduced for a sub-population of Lys63-linked di-ubiquitin from small-angle X-ray scattering data in solution.^6^ For Lys11-linked di-ubiquitin, chains in a single asymmetric unit of crystal structure display substantial variations with root mean square (rms) variations over 6 Å (Figure S1b).^8^

In a quest to understand what affords the multitude of quaternary structures and to elucidate a linkage–structure relationship for poly(ubiquitin), we serendipitously discovered that free ubiquitin dimerizes noncovalently in solution. At increasing protein concentration, a subset of peaks of ubiquitin shift progressively (Figure 1a and S2), corresponding to residues 8, 13, 44, 45, 46, 49, 67, 68, 70, 71, and 73, which are located at the β-sheet region of the protein and form a contiguous surface (Figure 1b). Plotting the chemical shift values measured at 30 °C over protein concentrations, the curves can be globally fit to a monomer–dimer equilibrium with a dissociation constant *K*_D_ of (4.9±0.3) mm (Figure 1c). Thus, the dimer population amounts to 50 % at approximately 5 mm protein concentration, and approximately 24 % (0.12 mm for dimeric species) at 1 mm. Backbone ^15^N relaxation rates are functions of the protein rotational correlation time *τ*_c_, which is in turn determined by the proportion of the dimer. The afforded *τ*_c_ values increase monotonously with protein concentration and can be fit to the same monomer–dimer equilibrium (Figure 1d). Further experimental evidence is provided by analytical ultracentrifugation analysis. Sedimentation equilibrium data collected at different wavelengths and different protein concentrations deviate from the curves predicted for a pure monomeric species, and give an apparent molecular weight that is approximately 20 % larger than the actual value. The best fit is obtained using a monomer–dimer equilibrium model with a *K*_D_ of (4.4±1.5) mm (Figure 2). Taken together, free ubiquitin molecules dimerize noncovalently in solution.

**Figure 1:**
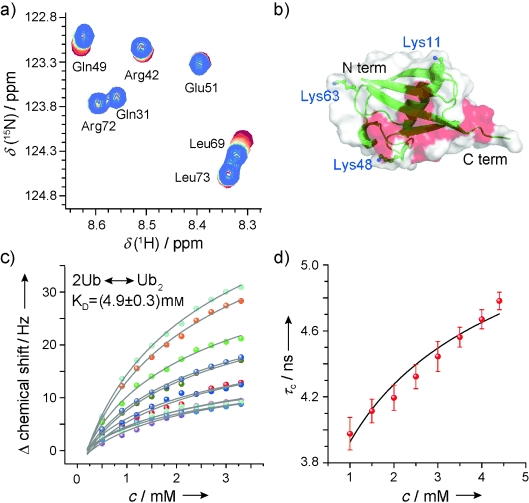
a) A representative region of 2D ^1^H-^15^N HSQC spectra of ^15^N-labeled ubiquitin, collected at concentrations from 0.2 mm to 3.3 mm (rainbow-colored from red to purple, respectively) at 30 °C; b) cartoon and surface representation of ubiquitin. Residues displaying large chemical shift changes (Δ*ω*_max_≥25 Hz) are colored red. Lys11, Lys48, and Lys63 are shown as ball-and-stick representations; c) changes of chemical shift values over protein concentrations for all perturbed residues can be globally fitted to a monomer–dimer equilibrium with *K*_D_=(4.9±0.3) mm. Relative to the lowest protein concentration (0.2 mm), the chemical shift differences are expressed as (*δ*_H_+δ_N_)^1/2^, in which *δ*_H_ and δ_N_ are in Hz; d) protein rotational correlation time *τ*_c_ can be fitted to the same equilibrium. Error bars represent one standard deviation.

**Figure 2:**
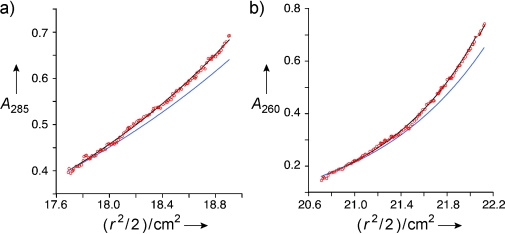
Sedimentation equilibrium analysis of ubiquitin: a) on a 0.8 mm sample with a rotor speed of 20 000 rpm, and b) on a 0.4 mm sample with a rotor speed of 32 000 rpm. The curves can be globally fitted to a monomer–dimer equilibrium model, with *K*_D_=(4.4±1.5) mm. The experimental data are shown as red circles, the fitted curves as black lines. The theoretical sedimentation curves expected for pure ubiquitin monomers are shown as blue lines.

To further characterize such a noncovalent dimer, we used paramagnetic relaxation enhancement (PRE), an NMR technique that affords long-range distance information for lowly populated species.10a,b A paramagnetic probe was attached to one of three engineered surface-exposed cysteines, K11C, K48C, and K63C, which are located at the periphery of the dimer interface mapped by chemical shift perturbation (Figure 1b). PRE NMR is extremely sensitive to the lowly populated species,11a–c in this case, the transiently formed noncovalent dimer of free ubiquitin. For a rapidly exchanging system, the observed PRE ^1^H-Γ_2_ rates become population-weighted average of PRE rates experienced in all conformational states.^12^ Providing that the paramagnetic center and the nuclei under investigation are closer to each other in the minor state, the transient species can be manifested through its disproportionally large contributions to the observed PRE. Applying the PRE technique, a number of weak protein oligomers have been previously characterized.^13^, 14a,b

Thus we measured the intermolecular PRE rates for the backbone amide protons of ^15^N-labeled wild-type ubiquitin mixed with equimolar unlabeled ubiquitin conjugated with a maleimide-EDTA-Mn^2+^ probe (Figure S3). The measured PRE rates arise exclusively from the paramagnetically tagged protein to the isotopically labeled protein, as a result of an ^15^N isotope filter in the NMR pulse sequence.^15^ With 100 mm NaCl in the solution, nonspecific protein–protein interactions would be largely suppressed.16a,b Owing to the flexibility of the paramagnetic tag, the paramagnetic center samples a rather large conformational space. Yet, the separations between conjugation sites (K11C, K48C, and K63) are much larger than the variations of the paramagnetic center (Figure S3), hence the intermolecular PREs from the three sites afford complementary observations. Interestingly, the PRE profiles for K11C, K48C, and K63C sites appear quite similar, with residues 12–14, 46–49, 71–76 displaying PRE Γ_2_ rates greater than 20 s^−1^ (Figure 3). These residues are located at the periphery of the dimer interface mapped by chemical shift perturbation (Figure 3 insets), indicating the two types of NMR data are consistent with one other.

**Figure 3:**
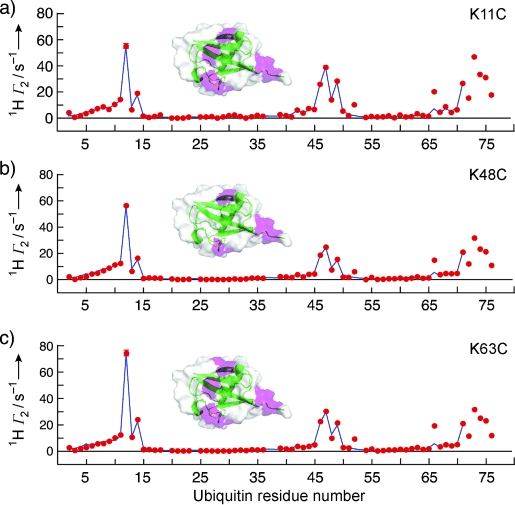
Intermolecular ^1^H transverse PRE G2 rates measured on an equimolar mixture (0.5 mm each) of ^15^N-labeled wild-type ubiquitin and unlabeled ubiquitin mutant (K11C, K48C, or K63C) conjugated with a maleimide-EDTA-Mn^2+^ probe. Back-calculated PRE rates for residues 1–71 are shown as blue lines. Insets: residues with observed PRE values >20 s^−1^ are colored purple on protein surface. Error bars represent one standard deviation.

To obtain an ensemble structure of the ubiquitin dimer, we performed rigid-body simulated annealing calculations by refining against the intermolecular PRE rates. The rigid body includes only residues 1–71, as the C-terminal residues 72–76 are flexible at ps–ns timescale.^17^ At the protein concentration employed for PRE experiments (1 mm total), 12 % of hetero-dimer was expected, which was used as a scaling factor for PRE back-calculation. The target function comprises the intermolecular PRE restraints for all three tagging sites, van der Waals repulsive term and a weak radius-of-gyration term applied to the entire dimer and to the dimer interface mapped by chemical shift perturbation.^18^ While keeping one subunit fixed, the other subunit is allowed to rotate and translate. Good agreement between observed and calculated PRE values is only achieved with multiple-conformer representation for the ubiquitin dimer. The overall PRE Q-factor is above 0.5 with three-conformer representation and levels off with 10 or more conformers (Figure 4a). Using a 10-conformer representation, the PRE Q-factors are 0.173, 0.191, 0.160, and 0.176 for all three sites, K11C, K48C, and K63C, respectively (Figure 4b), and the PRE profiles can be mostly reproduced (Figure 3a–c).

**Figure 4:**
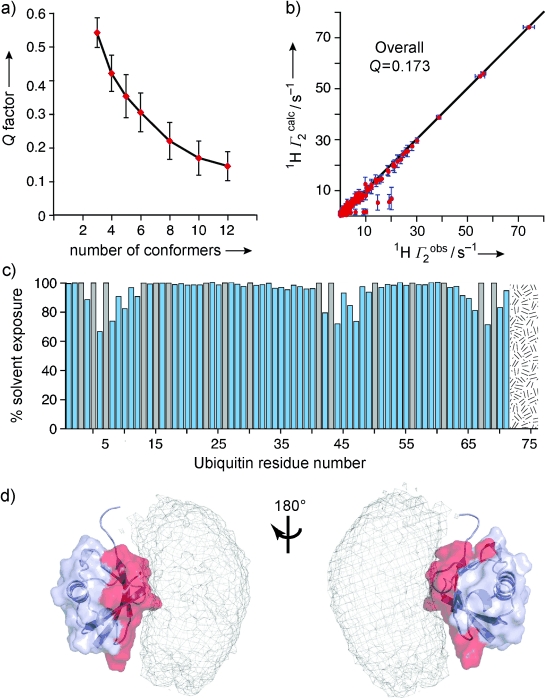
Ensemble structure of the noncovalent ubiquitin dimer; a) the PRE Q factors for all three tagging sites as a function of the number of conformers in the ensemble; b) correlations between observed and calculated PRE values, error bars represent one standard deviation; c) decrease in the relative solvent exposure upon dimerization; residues that are completely buried in the free form (solvent accessible area <10 Å) are denoted with a gray bar; d) reweighed atomic probability map plotted at 20 % threshold for the distribution of the ^15^N-labeled ubiquitin (gray meshes) relative to the unlabeled, paramagnetically tagged ubiquitin (purple surface). The two perspectives are related by an 180° rotation. The dimer interface is colored in red, encompassing residues 4–12, 42–51, and 62–71.

Upon forming a noncovalent dimer, the two ubiquitin molecules bury on average a solvent-accessible area of (601.8±336.5) Å, which encompasses residues 4–12, 42–51, and 62–71, all located at the β-sheet region of the protein (Figure 4c). The two subunits in the dimer are not confined to a single configuration but rather adopt an array of relative orientations (Figure 4d and S4). Ensemble distributions comprising a wide range of conformers have also been observed for other weak oligomers,14a,b which may be inherent to all weakly interacting systems.^19^ Importantly, the ensemble structure of ubiquitin dimers can be cross-validated against the intermolecular PRE data measured using a different paramagnetic probe conjugated at the same three sites (Figure S5a-b). Optimizing only the positions of nitroxide spin radical probes while fixing the dimer structure obtained from EDTA–Mn^2+^ PRE data, the back-calculated PRE values agree well with the observed values using the second paramagnetic probe (Figure S5c-e).

To the best of our knowledge, this is the first report showing the noncovalent dimerization of free ubiquitin; all previous work was focused on covalently linked poly(ubiquitin) or ubiquitin complexes with various UBDs. Noncovalent dimerization of free ubiquitin has several implications. First, a covalent linkage would promote noncovalent interactions. Although the concentration of free ubiquitin is only approximately 10 μm in mammalian cells,1a the protein mostly exists in covalently linked forms, affording a much higher effective concentration. For Lys48-linked di-ubiquitin, given that the linker consists of the C-terminal tail of the proximal unit (residues 72–76) and Lys48 side chain of the distal unit, the effective protein concentration for noncovalent dimerization is as high as (83.7±36.7) mm (Figure S6). As extrapolated from the dimerization equilibrium for the free ubiquitin, (83.9±1.9) % of the di-ubiquitin should be committed to closed conformation with the β-sheet region in contact, which is consistent with earlier experimental results.^9^

Secondly, a covalent linkage may restrict the relative movement for the two adjacent subunits in a poly(ubiquitin), and select a subset of conformers from the ensemble structure of the non-covalent dimer. Indeed, the crystal structure of Lys48-linked di-ubiquitin falls into the boundary of the atomic probability map delineated by the noncovalent dimer (Figure 5a). The noncovalent dimer, however, encompasses more interfacial residues than the Lys-48 linked covalent dimer—residues 4–7, 10–12, and 62–66 that are part of the dimer interface in the former become solvent-exposed in the latter (Figure 5b). Interestingly, these residues are also involved in interactions with certain UBDs20a–d and may permit the initial latching in respective binding processes.

**Figure 5:**
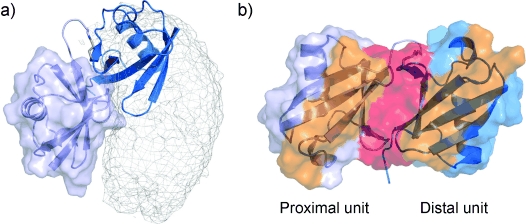
Comparison between the crystal structure of Lys48-linked di-ubiquitin and the ensemble structure of ubiquitin noncovalent dimer; a) the proximal unit (purple surface) of the di-ubiquitin crystal structure3a is superimposed to one subunit in the ensemble structure of the noncovalent dimer; the distal unit is shown as blue cartoon. The noncovalent dimer is represented the same way as in Figure 3; b) colored in orange, a large portion of the noncovalent dimer interface becomes exposed in Lys48-linked di-ubiquitin. The covalent dimer interface is colored in red.

Even though noncovalent ubiquitin dimerization appears compatible with every covalent linkage (Figure S4), it has been proposed that di-ubiquitins with Lys29, Lys33, Lys63 and head-to-tail linkages predominantly exist in open conformation with little inter-subunit contacts.^21^ A possible explanation is that steric hindrance around the amine group in the distal unit prevents the two linked subunits from coming together. Notwithstanding, it is likely that di-ubiquitin can fluctuate between open and closed conformations, albeit with different relative proportions for different linkage (Figure 6a). When in closed state, two adjacent subunits would still adopt multiple relative orientations (Figure 6b), but more restricted than the noncovalent dimer. Upon binding to a specific UBD, each ubiquitin subunit is molded to a particular tertiary structure, either by an induced-fit or conformational selection mechanism. As such, fluctuations among the ensemble members of ubiquitin dimer represent dynamics at the quaternary structure level that complements protein dynamics at the tertiary level.

**Figure 6:**
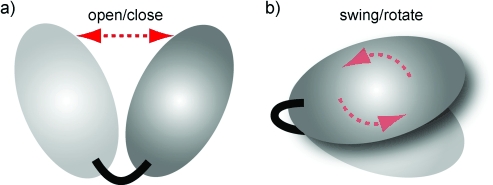
a) Scheme proposed for quaternary dynamics of di- or poly(ubiquitin); a) with an isopeptide linkage, the two adjacent ubiquitin subunits can either adopt open or closed conformations; b) when in a closed state, the two subunits can fluctuate among various relative orientations.

Ubiquitin has been a favorite model system for NMR and biophysics method development.23a–c Yet, the protein has always been assumed monomeric in solution. As the last implication but not the least, the presence of a noncovalent dimer for free ubiquitin, although minor, needs to be taken into account, especially when quantitating small differences.

## Supporting information

Detailed facts of importance to specialist readers are published as ”Supporting Information”. Such documents are peer-reviewed, but not copy-edited or typeset. They are made available as submitted by the authors.
