# Supplementary material for: Noncovalent Dimerization of Ubiquitin
Source: Angew Chem Int Ed Engl. 2011 Nov 23;51(2):469–72. doi: 10.1002/anie.201106190 (PMC3303887; doi:10.1002/anie.201106190)
Supplement: Supplementary file 1 [file anie0051-0469-SD1.pdf]

Angewandte Chemie
Eine Zeitschrift der Gesellschaft Deutscher Chemiker

Supporting Information

© Wiley-VCH 2011

69451 Weinheim, Germany

Noncovalent Dimerization of Ubiquitin**

*Zhu Liu, Wei-Ping Zhang, Qiong Xing, Xuefeng Ren, Maili Liu, and Chun Tang**

anie_201106190_sm_miscellaneous_information.pdf

Experimental Section

Sample preparation and NMR spectroscopy. K11C, K48C and K63C point mutations were introduced using QuikChange (Stratagene). Paramagnetic probes (maleimide-EDTA-Mn^{2+} and MTSL, from Toronto Research) were introduced as previously described.[1] NMR experiments were performed on a 800MHz Bruker instrument at 30°C in pH 6.0 10mM sodium acetate buffer containing 100mM NaCl. The intermolecular PRE values were acquired with two-point pulse sequence.[2] Backbone ^{15}N relaxation rates were measured using the standard pulse sequence. Analysis of the spectral density function afforded protein rotational correlation time τ_c.[3] Non-linear regression of the curves was performed with Microcal Origin 8.1. Fitting routine of monomer-dimer equilibrium was adapted from reference.[4]

Ensemble refinement of the dimer structure. There are two configurations when a thioether bond is formed between cysteine side chain and the maleimide-EDTA-Mn^{2+} probe. Thus, a six-conformer representation (three for each covalent configuration) is employed for each conjugation site. The MTSL spin radical probe conjugated via a disulfide bond was represented by three conformers.[5] Coordinates for ubiquitin were taken from PDB structure 1UBQ.[6] Rigid-body (ubiquitin residues 1-71) refinement using Xplor-NIH[7] were performed with a target function comprising the intermolecular PRE restraints for all three tagging sites, van der Waals repulsive term and a weak radius-of-gyration restraint[8] applied to the entire dimer and to the interfacial residues mapped by chemical shift perturbation. While keeping one subunit fixed, an ensemble of the other subunit (N_e=3-12) is allowed to rotate and translate. Calculated from the ubiquitin dimerization K_D, the population of the hetero-dimer formed between ^{15}N-labeled ubiquitin and unlabeled, paramagnetically tagged ubiquitin at 1mM total concentration (0.5mM each) is 12%, which is used as a scaling factor for PRE back-calculations. The refinement protocol for simulated annealing has been previously described.[9] The agreement between observed and calculated PRE rates are measured by the PRE Q-factor, defined as

$$Q_{PRE} = \left\{ \sum_i \left[\Gamma_2^{obs}(i) - \left\langle \Gamma_2^{calc}(i) \right\rangle \right]^2 / \sum_i \Gamma_2^{obs}(i)^2 \right\}^{1/2}$$

in which $\langle \Gamma_2^{Calc}(i) \rangle$ is the calculated PRE value for each residue averaged over 50 conformers, which were selected based on their lowest PRE and van der Waals energies. Atomic probability density map[10] and solvent accessible area were analyzed with Xplor-NIH. Interfacial analysis of ubiquitin-UBD complex structures was performed at PROTORP server at the Sussex University (http://www.bioinformatics.sussex.ac.uk/protorp). Structural figures were illustrated using PyMol.[11]

Analytical ultracentrifugation. Sedimentation equilibrium experiments were performed on a Beckman Coulter XL-A analytical ultracentrifuge. Protein samples were dialyzed against 10mM pH 6.0 sodium acetate buffer containing 100mM sodium chloride, and were then loaded into six-sector, 12-mm charcoal-filled Epon centerpieces. The centrifuge was run at speeds of 20,000 and 32,000 r.p.m. at 20°C, and scans were made every 2 hours at 0.001cm spacing with 20 replicates per point. Program Sedifit[12] was used to compare two consecutive scans and to assess whether the system reached equilibrium; solvent density and viscosity and protein reduced molecular weight σ was calculated using program Sednterp;[13] data were globally fit with program WinNONLIN.[14] Extinction coefficients at 260 and 285nm were calculated at 1057 and 1141.5 /cm/M, respectively, by referencing the absorbance to that of 280nm (ε_{280nm} = 1490/cm/M). Fitting to a single species yielded an apparent molecular weight of 10202.6±853.2, compared to 8564.9 Dalton, the actual molecular weight of ubiquitin. Lower square root of variance was afforded by fitting the data to a monomer-dimer equilibrium model. Absorbance-based monomer-dimer equilibrium constant $K_{2,abs}$ was converted to molar-based dimer dissociation constant K_D using equation $K_D = \left(K_{2,abs}(\varepsilon l)/2\right)^{-1}$, where l is the path length of the cell (1.2 cm) and ε is the molar extinction coefficient at the measured wavelength. The theoretical sedimentation curves for pure ubiquitin monomer were calculated using the following equation, where C_0 is the monomer absorbance at the fist data point r_0, and C_r is the absorbance at radial position r.

$$C_r = C_0 \exp\left[\sigma\left(\frac{r^2}{2} - \frac{r_0^2}{2}\right)\right]$$

Supporting Figures

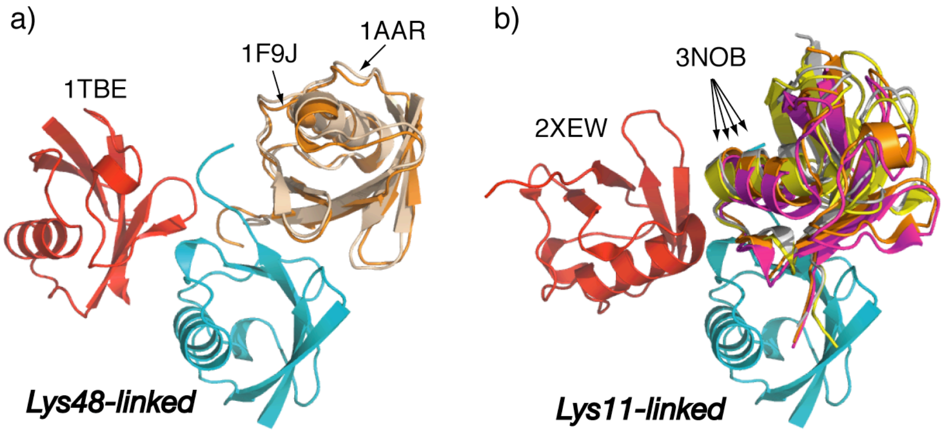

Figure S1. Crystal structures of di-ubiquitins with covalent linkages at a) Lys48 and b) Lys11. One subunit is superimposed (cyan), and the other is colored differently. Corresponding PDB codes are labeled. For Lys11-linked di-ubiquitin (PDB code 3NOB),[15] there are four molecules (8 chains) in each asymmetric unit. When superimposing one subunit, the r.m.s. differences for Cα atoms of the other subunit are over 6Å.

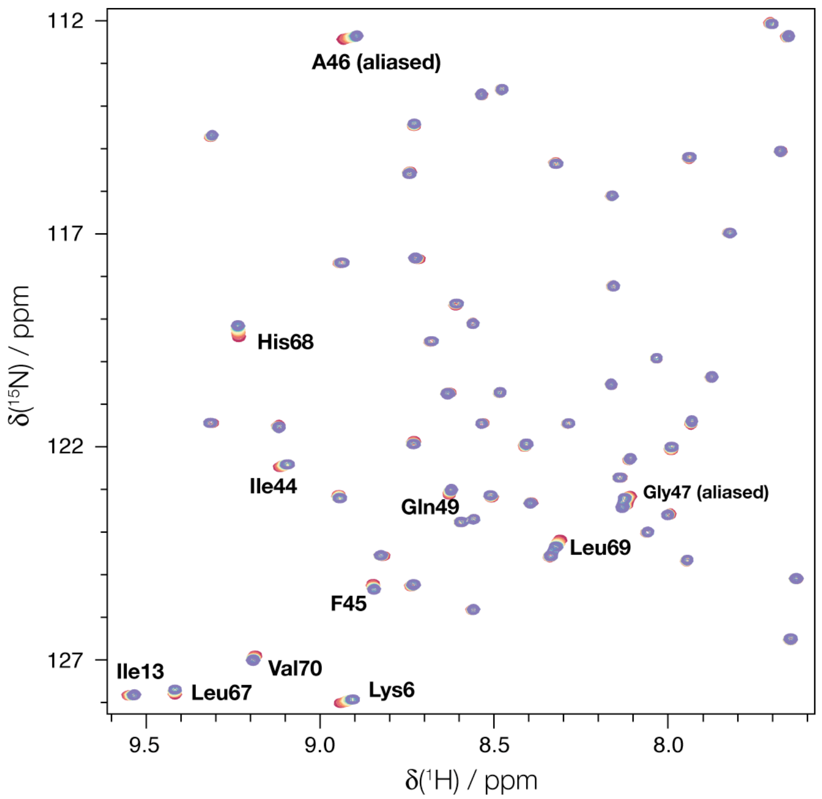

Figure S2. Overlay of two-dimensional ^{1}H-^{15}N spectra collected for ^{15}N-labeled ubiquitin at concentrations of 0.2, 0.5, 0.9, 1.2, 1.5, 1.8, 2.1, 2.4, 2.7, 3.0 and 3.3 mM, rainbow-colored from red to purple. Residues that display large chemical shift differences at increasing protein concentrations ($\Delta\omega_{max}\geq 25$Hz) are denoted.

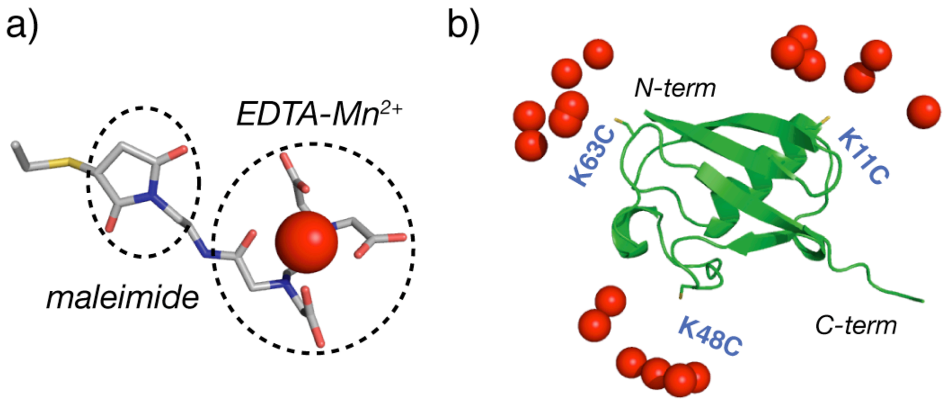

Figure S3. Maleimide-EDTA paramagnetic probe. a) Stick representation of the probe with Mn^{2+} shown as a red sphere. There are two configurations when a thioether bond is formed between the cysteine side chain and maleimide group, as the thiol group can attack from either side of the ring;[16] only one configuration is shown. (b) A representative ensemble distribution for the maleimide-EDTA-Mn^{2+} probe conjugated at K11C, K48C and K63C sites of ubiquitin. Red spheres indicate the possible positions of the metal. A six-conformer representation (three for each configuration) is employed for each conjugation site. Note that the separations between the tags are much larger than the variations of the paramagnetic center.

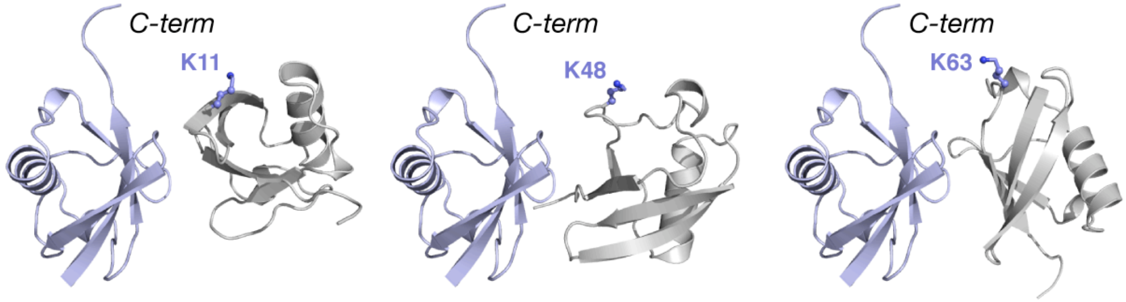

Figure S4. Representative conformers from the ensemble structure of ubiquitin non-covalent dimer, showing various orientations between the two subunits. Giving the flexibility of the covalent linker, these conformers appear compatible with respective covalent linkage; the side chains of Lys11, Lys48 and Lys63 are shown.

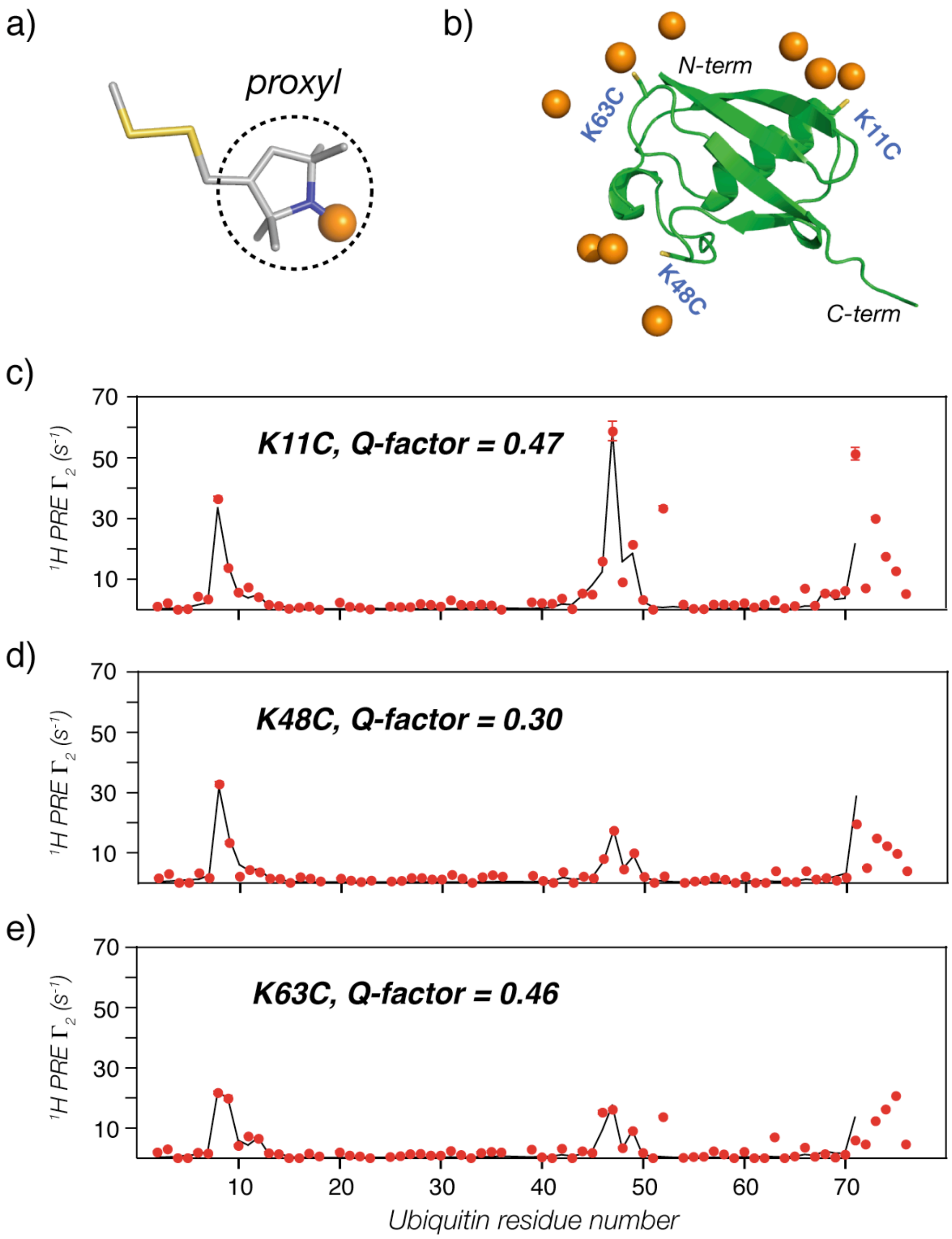

Figure S5. Cross-validation of the ensemble structure of non-covalent ubiquitin dimer. (a) The structure of MTSL probe, a five-membered proxyl ring of nitroxide radical, is conjugated to protein thiol group via a disulfide bond, with the oxygen atom of the spin radical shown as orange spheres. (b) A three-conformer representation for the spin radical probe conjugated at K11C, K48C and K63C of ubiquitin. (c-e) PRE profiles for K11C, K48C and K63C measured with spin radical probe. The observed values are denoted as red spheres, and the black lines indicate back-calculated values. PRE values for residues 1-71 are calculated, and the corresponding Q-factors are given. Error bars represent 1 standard deviation.

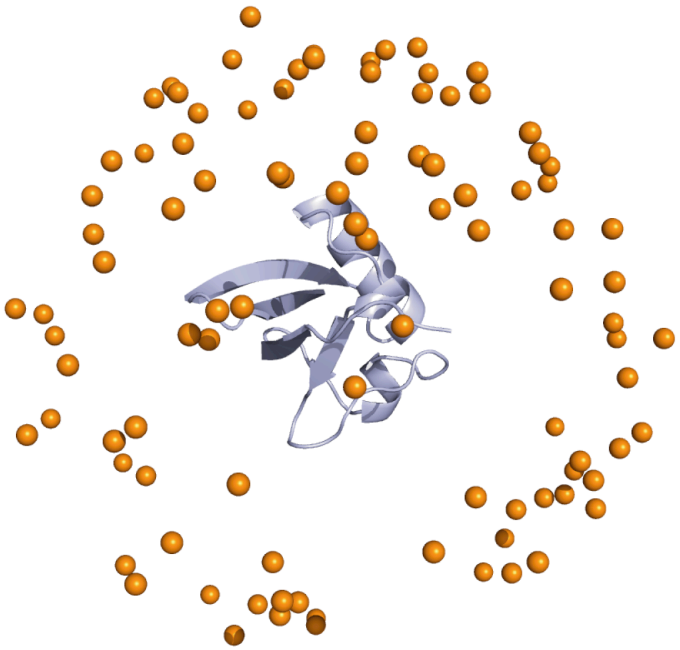

Figure S6. Estimation of the effective protein concentration for non-covalent dimerization for Lys48-linked di-ubiquitin. Subject to only van der Waals repulsive term, the torsional angles for the covalent linker between the two subunits comprising the C-terminal tail of the proximal unit (residues 72-76) and the lysine side chain of the distal unit are randomized, affording an average center-of-mass distance 33.6±3.9 Å between the two linked subunits. Illustrated here, the proximal unit is shown as light purple cartoon, and the centers of mass for 100 distal units with randomized positions are shown as orange spheres. The spherical volume that the distal unit occupies is 19848±8700Å^3. Using the Avogadro's constant, the effective concentration of the distal unit is 83.7±36.7mM. Errors are derived from propagation of uncertainties.

[1] C. Tang, J. Iwahara, G. M. Clore, *Nature* **2006**, *444*, 383-386.

[2] J. Iwahara, C. Tang, G. M. Clore, *J. Magn. Reson.* **2007**, *184*, 185-195.

[3] L. E. Kay, D. A. Torchia, A. Bax, *Biochemistry* **1989**, *28*, 8972-8979.

[4] J. S. Chen, R. B. Shirts, *J. Phys. Chem.* **1985**, *89*, 1643-1646.

[5] J. Iwahara, C. D. Schwieters, G. M. Clore, *J. Am. Chem. Soc.* **2004**, *126*, 5879-5896.

[6] S. Vijay-Kumar, C. E. Bugg, W. J. Cook, *J. Mol. Biol.* **1987**, *194*, 531-544.

[7] C. D. Schwieters, J. J. Kuszewski, N. Tjandra, G. M. Clore, *J. Magn. Reson.* **2003**, *160*, 65-73.

[8] C. Tang, G. M. Clore, *J. Biomol. NMR* **2006**, *36*, 37-44.

[9] C. Tang, R. Ghirlando, G. M. Clore, *J. Am. Chem. Soc.* **2008**, *130*, 4048-4056; C. Tang, J. M. Louis, A. Aniana, J. Y. Suh, G. M. Clore, *Nature* **2008**, *455*, 693-696.

[10] C. D. Schwieters, G. M. Clore, *J. Biomol. NMR* **2002**, *23*, 221-225.

[11] W. L. Delano, *DeLano Scientific, Palo Alto, CA, USA* **2002**.

[12] P. Schuck, *Biophys. J* **2000**, *78*, 1606-1619.

[13] J. S. Philo, *Biophys. J* **1997**, *72*, 435-444.

[14] D. Yphantis, M. L. Johnson, J. W. Lary, **1997**, WinNONLIN106 program (National Analytical Ultracentrifugation Facility, Univ. Connecticut, Storrs, CT).

[15] A. Bremm, S. M. Freund, D. Komander, *Nat. Struct. Mol. Biol.* **2010**, *17*, 939-947.

[16] C. Tang, C. D. Schwieters, G. M. Clore, *Nature* **2007**, *449*, 1078-1082.
